# Survival results of 3786 patients with stage I or II laryngeal squamous cell carcinoma: a study based on a propensity score

**DOI:** 10.1016/j.bjorl.2020.06.007

**Published:** 2020-07-19

**Authors:** Genival Barbosa de Carvalho, Hugo Fontan Kohler, Renan Bezerra Lira, José Guilherme Vartanian, Luiz Paulo Kowalski

**Affiliations:** a A.C. Camargo Cancer Center, Departamento de Cirurgia de Cabeça e Pescoço, São Paulo, SP, Brazil; b A.C. Camargo Cancer, Centro de Referência de Tratamento dos Pacientes com Tumores de Cabeça e Pescoço, São Paulo, SP, Brazil; c Universidade de São Paulo, Faculdade de Medicina, Departamento de Cirurgia de Cabeça e Pescoço e LIM 28, São Paulo, SP, Brazil

**Keywords:** Laryngeal cancer, Surgery, Radiotherapy, Prognostic factors

## Abstract

**Introduction:**

Laryngeal squamous cell carcinoma is the second most prevalent malignancy among head and neck tumors, and the treatment of patients with stage I or II disease can be performed with surgery or radiation therapy. National population studies describing therapeutic results comparing these modalities are unusual, but they can be very important to direct treatment guidelines.

**Objective:**

To evaluate the survival results of patients with laryngeal squamous cell carcinoma at clinical stages I or II, according to the main therapeutic modalities used.

**Methods:**

Cross-sectional, population-based study using the database of Fundação Oncocentro de São Paulo from January 2000 to March 2019. Inclusion criteria were patients with laryngeal squamous cell carcinoma in clinical stages cT1-2N0. To compensate for the non-random allocation of patients and the imbalance between confounding variables between groups, we used the propensity score methodology.

**Results:**

A total of 3786 patients met the inclusion criteria. Regarding the cT stage, there were 2171 patients (57.3%) with cT1 tumors. Patients in the public health system had a longer time between diagnosis and treatment (*p* < 0.001). The analysis by propensity score showed that patients treated with surgery had a tendency towards better disease-specific survival (*p*  = 0.012). Comparing radiotherapy alone *versus* its combination with radiochemotherapy, radiotherapy alone showed a tendency towards a better survival rate (*p* < 0.001).

**Conclusion:**

Analysis by propensity score identified better results for disease-specific survival in patients with laryngeal squamous cell carcinoma at clinical stages I and II treated by surgery when compared to radiotherapy.

## Introduction

Of upper airway carcinomas, the laryngeal carcinoma is the second most common in the world.^1^ Brazil, according to the National Cancer Institute (INCA, *Instituto Nacional do Câncer*) has one of the highest incidence rates in the world, with 5.7 cases per 100,000 men and the estimate of new cases in 2020 was 6470, of which 1980 in the state of São Paulo.^2^

The study by Carvalho et al. demonstrated a significant decrease in the overall survival rates for patients with laryngeal tumors diagnosed in early stages, from 82.3% in 1985 to 74.3% in 1997, according to an analysis of the American database, “Surveillance, Epidemiology and End Results” (SEER).^3^ The latest SEER data from 2009 to 2015 recorded a 5-year survival rate of 77% for localized disease and a mean survival rate of 60.3%; in 2011, for all stages (SEER).^4^

In patients with stage I or II disease, treatment is generally unimodal, consisting of radiotherapy (RT) or surgery, with similar overall survival results.^5^, ^6^, ^7^, ^8^, ^9^, ^10^, ^11^ The prospective, randomized study that compared surgery and radiotherapy in the treatment of 234 patients with early laryngeal cancer did not find any significant differences in overall or cancer-specific survival rates between the two therapeutic modalities. In patients with stage cT1N0 tumors submitted to radiotherapy, the overall survival (OS) in 5 years was 91.7% and 100% in patients submitted to surgery. In patients with cT2N0 stage tumors, 5-year survival rates of 88.8% were recorded in patients undergoing radiotherapy and 97.4% in those undergoing surgical treatment. Moreover, patients submitted to radiotherapy had higher rates of local recurrence.^8^ Despite being prospective and randomized, this study had important methodological flaws: the groups were not balanced, the staging criteria were inadequate, the treatment performed was not the same in the different institutions and the time of follow-up was short.^9^, ^10^

The meta-analysis published by Guimarães et al., which compared endoscopic surgery with radiotherapy for the treatment of glottic cancer at cTis/T1aN0 stage, found better overall cancer-specific survival rates for patients undergoing transoral laser surgery (TOLS).^11^ The study by Jones et al., with 488 patients with clinical stage I or II laryngeal tumors, did not observe significant differences in the rates of overall cancer-specific survival or local recurrence-free between patients undergoing surgery in relation to radiotherapy, but patients undergoing surgery showed higher rates of regional recurrence.^5^ The heterogeneity of treatment details, the different periods of treatment, the study designs and the evaluation of the two therapeutic modalities are important limitations of these studies. The small number of treatment failures and local variations in treatment preferences by physicians and patients have frustrated efforts to perform new randomized trials comparing these therapeutic modalities.^12^

Although the survival results of patients with laryngeal squamous cell carcinoma (SCC) at clinical stages cT1N0 or cT2N0 have been widely studied, population studies describing the therapeutic results comparing these modalities are still rare, but they can be very important to guide public policy choices. This study aims to evaluate the survival results of patients with stage I or II laryngeal neoplasia treated in the state of São Paulo, consistent with data from Fundação Oncocentro de São Paulo (FOSP), according to the therapeutic options used. The hypothesis to be evaluated is that patients undergoing initial treatment with radiotherapy have worse cancer-specific survival.

## Methods

This is a cross-sectional, population-based study that used the database of FOSP. This database comprises data sent by the Hospital Cancer Registry. Data collection started on January 1, 2000. Only cases without prior treatment are considered and their followup data are updated quarterly. The database used in the study was downloaded from the FOSP website (www.fosp.saude.sp.gov.br) on 5/1/2019 and refers to the period from January 2000 to March 2019.^13^

Inclusion criteria were patients with C32 topography primary tumors (larynx) at clinical stage cT1-2 cN0 and a morphological diagnosis of squamous cell carcinoma.

The variables studied were age, gender, clinical stage and type of treatment and the outcomes were overall and cancer-specific survival. The statistical analysis was performed using the Stata 15 (Stata Corp, College Station — TX, USA) and R (www.cran.r-project.org) statistical package. The categorical variables are described as frequencies, while continuous variables are described as mean and standard deviation (SD). The survival analysis was performed initially by calculating the Kaplan–Meier estimator, followed by the Cox model for univariate comparison between treatments. To compensate for the non-random allocation of patients and the imbalance between confounding variables between groups, we used the propensity score methodology. The pairing was performed in a one-to-one ratio using a variation of 0.05 multiplied by the SD of the logit model. After using the propensity score, the analysis was repeated only with the treatment type variable.

## Results

A total of 3786 patients met the inclusion criteria. There were 3339 men (88.2%) and 447 women (11.8%). The age at diagnosis ranged from 20 to 103 years (mean, 63.0 years and SD, +10.5 years). As for the primary tumor site of origin, 1769 (46.7%) were glottic, 445 (11.8%) supraglottic, 25 (0.7%) infraglottic and 1547 (40.9%) had an unspecified location (ICD C32.8 and C32.9). As for the cT stage, there were 1371 cT1 tumors (36.2%), 536 (14.1%) cT1a, 264 (6.9%) cT1b and 1615 (42.6%) cT2 tumors (Table 1).

**Table 1:** Distribution of the cT stage according to the primary tumor topography.

Topography	cT1	cT1a	cT1b	cT2	Total
C32.0	596	432	198	543	1769
C32.1	139	13	4	289	445
C32.2	12	0	0	13	25
C32.8 + C32.9	624	91	62	770	1547
Total	1371	536	264	1615	3786

**Figure 1:**
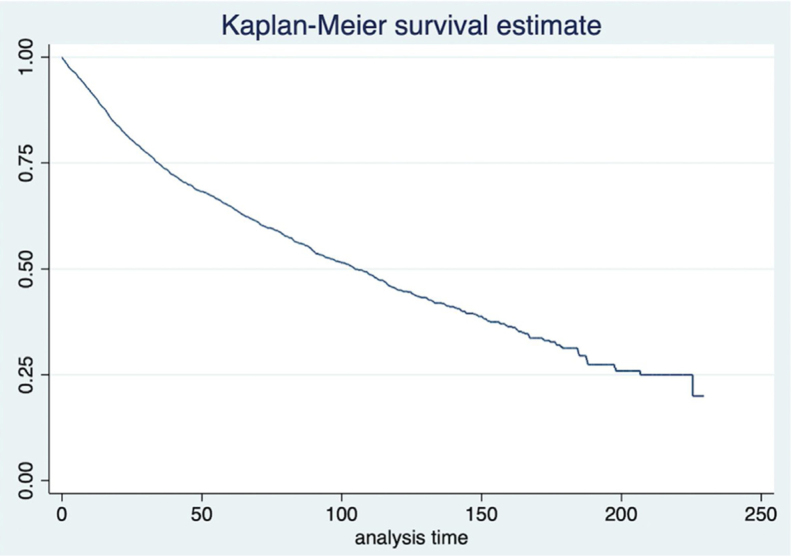
Overall survival.

**Figure 2:**
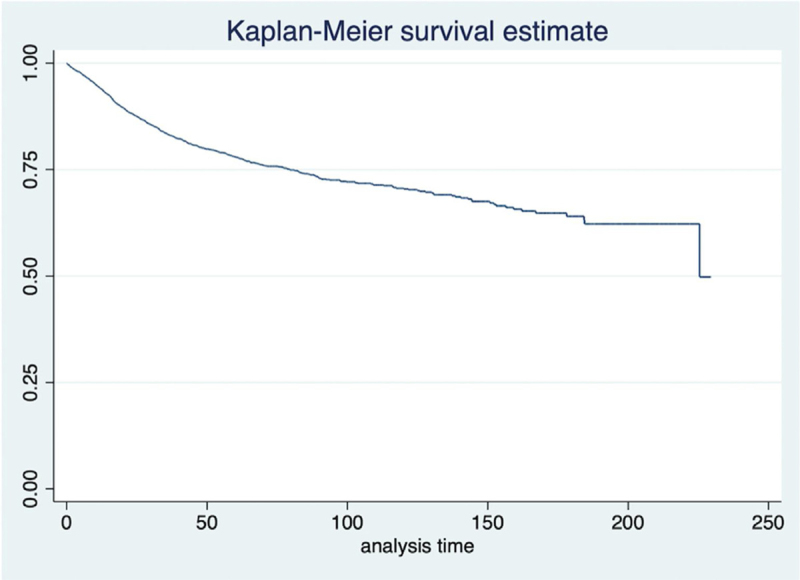
Cancer-specific survival.

The mean waiting time between diagnosis and treatment was 8.2 weeks, ranging from 3.2 weeks for private patients to 9.5 weeks for patients assisted by the supplementary system and 14.0 weeks for patients treated by the Unified Health System (SUS) (*p* < 0.001). The mean time of followup was 55.7 months and ranged from 1 day to 229.5 months. According to the last information, there were 441 living patients with cancer (11.6%), 1819 (48.1%) living patients without evidence of disease, 794 patients (21.0%) who died from cancer and 732 patients (19.3%) who died from other causes. The overall and disease-specific survival curves are shown in Figure 1, Figure 2, respectively.

The univariate analysis of survival showed age at diagnosis, gender, clinical stage and treatment modality as significant for disease-specific survival (Table 2). Using the same variables, the multivariate analysis of survival was performed (Table 3). Regarding the treatment modality, we made comparisons between the following subgroups: surgery *versus* radiotherapy, both exclusively, and radiotherapy *versus* the association of radiotherapy and chemotherapy. However, patients submitted to each therapeutic option had different demographic characteristics (Table 4).

**Table 2:** Univariate analysis of disease-specific survival in the database of *Fundação Oncocentro de São Paulo*.

Variable	Values	Hazard ratio	95%CI	*p*-Value
Age		1.015	1.008–1.023	<0.001

Gender	Male	1		
	Female	0.708	0.551–0.909	0.007

Clinical stage	I	1		
	II	1.843	1.591–2.135	<0.001

Treatment	Surgery	1		
	RT	1.323	1.074–1.629	0.009
	CT	7.138	4.426–11.512	<0.001
	Surgery + RT	1.305	1.007–1.693	0.044
	Surgery + CT	3.341	1.762–6.336	<0.001
	RT + CT	2.690	2.103–3.440	<0.001
	Surgery + RT + CT	2.873	2.140–3.859	<0.001
	Surgery + RT + CT + HT	NA		
	Other modalities	1.464	1.108–1.933	0.007
	Untreated	NA		

CI, confidence interval; RT, radiotherapy; CT, chemotherapy; HT, hormone therapy.

**Table 3:** Multivariate analysis of disease-specific survival in the database of *Fundação Oncocentro de São Paul*o.

Variable	Values	Hazard ratio	95%CI	*p*-Value
Age		1.017	1.009–1.025	<0.001

Gender	Male	1		
	Female	0.656	0.511–0.844	0.001

Clinical stage	I	1		
	II	1.666	1.430–1.941	<0.001

Treatment	Surgery	1		
	RT	1.284	1.041–1.584	0.019
	CT	6.280	3.886–10.149	<0.001
	Surgery + RT	1.279	0.986–1.659	0.064
	Surgery + CT	2.923	1.540–5.548	0.001
	RT + CT	2.338	1.820–3.002	<0.001
	Surgery + RT + CT	2.597	1.929–3.496	<0.001
	Surgery + RT + CT + HT	NA		
	Other modalities	1.357	1.026–1.793	0.032
	Untreated	NA		

CI, confidence interval; RT, radiotherapy; CT, chemotherapy; HT, hormone therapy.

**Table 4:** Demographic characteristics of patients with squamous cell carcinoma according to the treatment modality.

		Radiotherapy alone	Surgery	Radiotherapy and chemotherapy
Gender	Female	117 (10.43%)	127 (11.56%)	41 (12.73%)
	Male	1.005 (89.57%)	972 (88.44%)	281 (87.27%)

Age		64.63 (10.67)	61.99 (10.12)	62.05 (10.35)

Paying source	SUS	452 (40.29%)	460 (41.86%)	158 (49.07%)
	Health insurance	66 (5.88%)	51 (4.64%)	5 (1.55%)
	Private	15 (1.34%)	18 (1.64%)	2 (0.62%)
	Not informed	589 (52.50%)	570 (51.87%)	157 (48.76%)

Topography	C32.0	656 (58.47%)	580 (52.78%)	90 (27.95 %)
	C32.1	104 (9.27%)	114 (10.37%)	73 (22.67%)
	C32.2	4 (0.36%)	8 (0.73%)	3 (0.93%)
	C32.8	17 (1.52%)	10 (0.91%)	9 (2.80%)
	C32.9	341 (30.39%)	387 (35.21%)	147 (45.65%)

cT stage	cT1	408 (36.36%)	445 (40.49%)	70 (21.74%)
	cT1a	191 (17.02%)	204 (18.56%)	13 (4.04%)
	cT1b	108 (9.63 %)	60 (5.46 %)	14 (4.35 %)
	cT2	415 (36.99%)	390 (35.49%)	225 (69.88%)

SUS, Brazilian Unified Health System (*Sistema Único de Saúde*).

**Figure 3:**
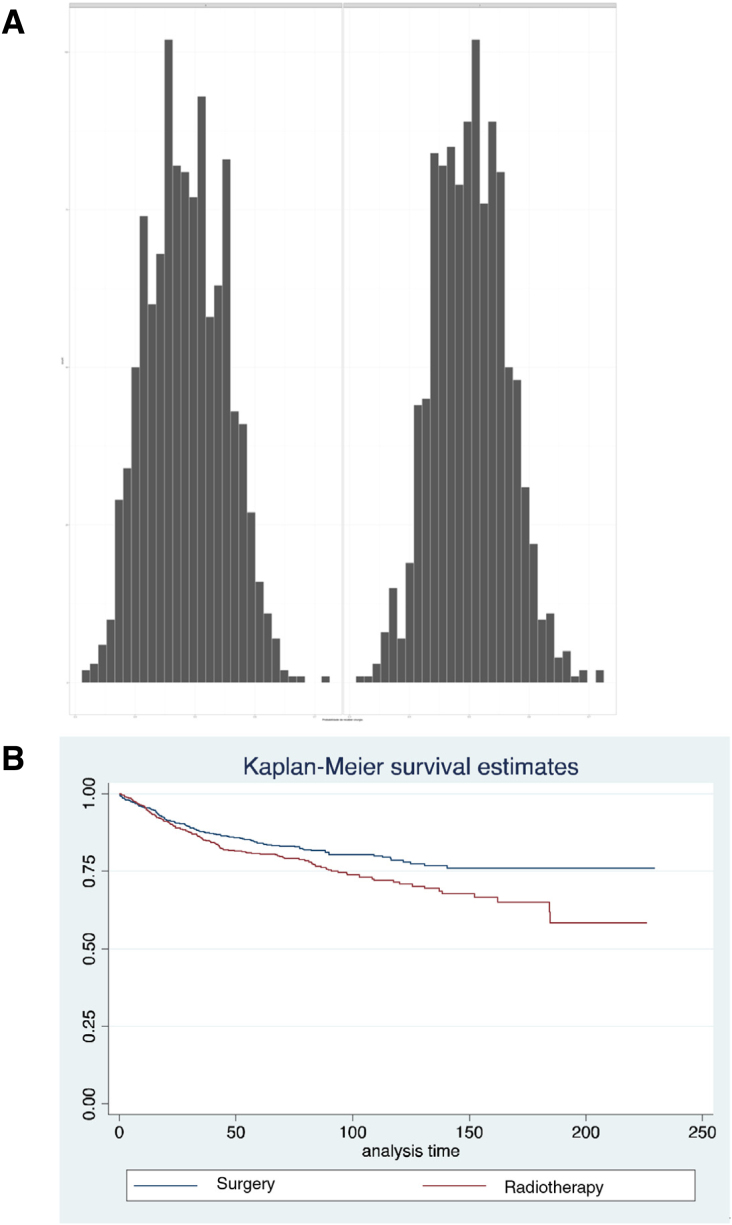
(A) Histograms of propensity scores in each of the treatment comparisons performed. (B) Survival after pairing of patients undergoing RT or surgery alone.

**Figure 4:**
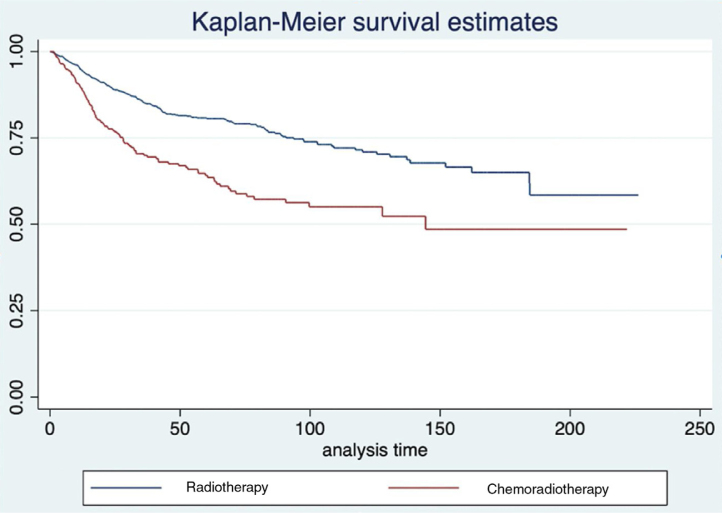
Survival after pairing of patients submitted to RT or chemoradiotherapy.

To compensate for biases in treatment indications and by the distribution of independent variables among the different groups, the survival analysis was repeated using propensity scores. The distribution of these scores for patients undergoing radiotherapy or surgery alone can be seen in Fig. 3a. In this situation, the pairing of 2056 patients was possible. After pairing, we observed a trend towards a lower disease-specific survival rate was observed in patients treated with radiotherapy (HR = 1.237; 95% CI 0.996–1.541; *p* = 0.012) (Fig. 3b). When comparing between radiotherapy alone and the radiochemotherapy association, we observed an important difference in the histogram of propensity scores (Fig. 3b), and the pairing of 642 patients was possible, in total. In the analysis of disease-specific survival, there is a significant advantage for radiotherapy alone, when compared to radiochemotherapy (HR = 1.476; 95%CI: 1.249–2.291; *p* < 0.001) (Fig. 4).

## Discussion

The aim of the study was to identify the factors that affect the prognosis of patients with early-stage laryngeal squamous cell carcinoma, with a trend towards a better disease-specific survival for female patients with tumors in clinical stage I and those undergoing surgery when compared to isolated radiotherapy being observed. Most of the patients who had information on the paying source were treated by SUS (49.07%), where the mean time between diagnosis and treatment was longer. This delay in treatment can negatively affect the prognosis and can occur either due to the lack of infrastructure to perform the surgery at the institution where the diagnosis was made, or due to difficulties in having access to radiotherapy treatment. The study by Kompelli et al. demonstrated that the need to change the institution for the adequate treatment of the patient results in a significant delay at the start of treatment, which worsens the prognosis and increases in costs.^14^

It was observed that patients treated with radiotherapy had an older median age and, proportionally, a higher prevalence of stage cT1b tumors, when compared to those submitted to surgery and thus, the propensity score was performed to try to reduce the impact of these selection biases on the assessed outcomes. This method has been shown to be a good option for prospective randomized studies.^15^, ^16^, ^17^ Even after pairing, with the correction of selection biases, patients undergoing treatment with radiotherapy had a risk of death from the disease that was approximately 28% higher when compared to those submitted to surgery. Although the prospective cited by Warner et al. (p.6) did not observe a statistically significant difference in relation to the overall cancer-specific survival rates for patients with stage cT1N0 or cT2N0 disease, when comparing surgery with radiotherapy, a higher rate of local recurrence was observed in patients undergoing radiotherapy.^8^ Although it was the only prospective randomized study found in the literature, important methodological flaws were observed, so the results of this study may have been compromised.^9^, ^10^

The treatment of patients with laryngeal SCC clinical stages I or II is traditionally unimodal: surgery or radiotherapy.^5^, ^6^, ^7^, ^8^, ^9^, ^10^, ^11^ The radiochemotherapy association has been best described for patients with stage III or IV laryngeal SCC. The study published by Wolf et al. included 31 patients (9.3%) with cT1 or cT2 stages and the RTOG 91−11 trial published by Forastiere et al. in 2003, included 33 (6.4%) patients with cT2 tumors; in both studies, these were patients with cervical metastases and candidates for total laryngectomy.^18^, ^19^ As it is a population-based study, it is not possible to understand the reasons why some patients were treated with therapeutic associations, but we observed that patients submitted to radiotherapy alone had better disease-specific survival than patients submitted to radiochemotherapy. The greater difficulty in having access to radiotherapy, due to the lack of services in Brazil,^20^ can lead to a worse prognosis due to the risk of disease progression. Moreover, another probable problem related to the worse prognosis of patients submitted to radiotherapy is staging errors. Patients submitted to surgery have a more accurate staging due to intraoperative findings, as demonstrated in the study by Locatello et al. in 2019, which compared the clinical and pathological staging of patients with laryngeal squamous cell carcinoma, showing that about 32% of the patients have differences between the clinical and the pathological staging. The disparity was more common in patients with glottic tumors when compared to those with tumors of supraglottic location (13.5% and 7.4%, respectively, *p*  = 0.519). Another interesting finding of the study was the observation that patients with local recurrence after the initial treatment with radiotherapy had worse overall and cancer-specific survival and an increased risk of developing second locoregional recurrences compared to patients with the same rTNM stages that had initially been treated by surgery.^21^

The patient’s clinical condition, the possibility of adequate followup and the experience of the multidisciplinary team in performing partial rescue laryngectomies contribute to a better therapeutic result, including the possibility of long-term laryngeal preservation in the subgroup of patients who developed local recurrence,^22^, ^23^, ^24^ as most patients with local recurrence after radiotherapy undergo total laryngectomy.^25^ Therefore, the initial therapeutic choice must also consider these factors and the results of rescue treatments. Even in cases eligible for partial laryngectomy, when performed for rescue treatment after failure of the initial radiotherapy treatment, these procedures require a longer time period until decannulation and removal of the nasoenteral tube^25^, ^26^ and show a higher rate of second new recurrences in this scenario.^21^

Considering the difficulties in having access to the Brazilian public health system, particularly for the beginning of radiotherapy, the difficulties for adequate followup and the fact that the majority of patients with local recurrence after radiotherapy are submitted to total rescue laryngectomy, surgery should be considered the initial treatment of choice, especially when radiotherapy is not equally accessible.

## Conclusion

Patients with clinical stages I and II laryngeal squamous cell carcinoma treated by surgery show better cancer-specific survival results when compared to radiotherapy after analysis by propensity score.

## Conflicts of interest

The authors declare no conflicts of interest.
